# Dynamical modelling of phenotypes in a genome-wide RNAi live-cell imaging assay

**DOI:** 10.1186/1471-2105-14-308

**Published:** 2013-10-16

**Authors:** Gregoire Pau, Thomas Walter, Beate Neumann, Jean-Karim Hériché, Jan Ellenberg, Wolfgang Huber

**Affiliations:** 1 EMBL, Heidelberg, 69117 Germany; 2 Department of Bioinformatics and Computational Biology, Genentech Inc., South San Francisco, California, 94080, USA; 3 Centre for Computational Biology, Mines ParisTech, Fontainebleau, 77300 France; 4 Institut Curie, Paris, 75248, France; 5 U900, INSERM, Paris, 75248, France

**Keywords:** High-throughput time-lapse imaging assay, siRNA screening, Mitosis regulation, Differential equation modelling

## Abstract

**Background:**

The combination of time-lapse imaging of live cells with high-throughput perturbation assays is a powerful tool for genetics and cell biology. The Mitocheck project employed this technique to associate thousands of genes with transient biological phenotypes in cell division, cell death and migration. The original analysis of these data proceeded by assigning nuclear morphologies to cells at each time-point using automated image classification, followed by description of population frequencies and temporal distribution of cellular states through event-order maps. One of the choices made by that analysis was not to rely on temporal tracking of the individual cells, due to the relatively low image sampling frequency, and to focus on effects that could be discerned from population-level behaviour.

**Results:**

Here, we present a variation of this approach that employs explicit modelling by dynamic differential equations of the cellular state populations. Model fitting to the time course data allowed reliable estimation of the penetrance and time of appearance of four types of disruption of the cell cycle: quiescence, mitotic arrest, polynucleation and cell death. Model parameters yielded estimates of the duration of the interphase and mitosis phases. We identified 2190 siRNAs that induced a disruption of the cell cycle at reproducible times, or increased the durations of the interphase or mitosis phases.

**Conclusions:**

We quantified the dynamic effects of the siRNAs and compiled them as a resource that can be used to characterize the role of their target genes in cell death, mitosis and cell cycle regulation. The described population-based modelling method might be applicable to other large-scale cell-based assays with temporal readout when only population-level measures are available.

## Background

High-throughput cell imaging assays allow broad and quantitative measurement of the response of cell populations to perturbations including drugs [1], small molecules [2] and small interfering RNA (siRNA) [3]. Screens have revealed genes whose depletion affects cell cycle progression [4], measured the effects of drugs on the morphology of HeLa cells [5] and identified novel DNA damage factors by grouping genes by phenotypic similarity [6]. Most screening experiments are performed as endpoint assays and provide observations that in many cases are consequences of unseen intermediate events. Thus, functional interpretation of results from endpoint analysis can be obscured by indirect effects. High-throughput time-lapse imaging is a technique [7] that overcomes this limitation and considerably extends the potential of biological discovery by capturing the dynamic aspects of the observed phenotypes. A typical feature of large-scale assays is that the range of observed phenotypes has multiple dimensions, reflecting for example the different effects of perturbations on cell growth, cytoskeleton structure, cell division or motility. A goal of the data analysis is the extraction of multivariate, but relatively low-dimensional phenotypic descriptors that are biologically meaningful, interpretable and robust to experimental noise. In the case of time-resolved data, the time-dependence of the observations needs to be appropriately described and summarised.

The Mitocheck project performed a time-lapse imaging assay that employed siRNAs to test the implication of human genes in transient biological processes such as cell division or migration genome-wide [8]. In this experiment, HeLa cells stably expressing core histone 2B tagged with green fluorescent protein (GFP) were seeded on siRNA-spotted slides, incubated for 18 h and imaged with automated fluorescence microscopy for 48 h. Video sequences of cell populations on each siRNA-spot were analysed by image segmentation, and at each frame, each individual cell was categorised into one of 16 morphological classes mostly related to cell division (Table 1). By comparing the abundances of the different morphological classes to negative control experiments, 1249 genes were identified as potential mitotic hits (“primary screen”). Subsequently, further validation experiments were done using independent siRNAs (“validation screen”) and rescue of 16 gene products using orthologous mouse genes.

**Table 1:** Nuclear morphology count statistics in the Mitocheck assay

**Mitocheck nuclear morphology**	**Number of cells**	**Percentage of total**	**mitoODE cellular state**
Interphase	1788193783	81.89	Interphase
Large	31803343	1.46	Interphase
Elongated	19680422	0.90	Interphase
Folded	14121061	0.65	Interphase
Hole	6374658	0.29	Interphase
SmallIrregular	18118093	0.83	Interphase
UndefinedCondensed	4266163	0.20	Interphase
Metaphase	12242280	0.56	Mitosis
Anaphase	44535322	2.04	Mitosis
MetaphaseAlignment	12854397	0.59	Mitosis
Prometaphase	17551608	0.80	Mitosis
ADCCM	4900034	0.22	Mitosis
Shape1	87196372	3.99	Polynucleated
Shape3	69356097	3.18	Polynucleated
Grape	5531756	0.25	Polynucleated
Apoptosis	27746493	1.27	Cell death
Artefact	19199683	0.88	
Total	2183671565	100.00	

The table shows the counts of the 16 nuclear morphologies (excluding the “Artefact” and “Total” rows) defined in the Mitocheck assay and the grouping into 4 cellular states described in our manuscript.

This analysis of the Mitocheck data generated an enormous wealth of results about the implication of human genes in cell division, but did not fully exhaust the information contained in the data. Several temporal features including the time of mitotic arrest, cell death or cell cycle arrest, or the duration of mitosis were not quantified. In principle, the nature of the data –time-lapse movies of dividing cells– asks for analysis of the single-cell tracking graphs [9,10]. However, reliable tracking of the cells used in this experiment requires a time resolution between image frames lower than 10 min. For the main Mitocheck data set, the decision had been made to use a lower temporal sampling frequency ((30 min)^-1^) in order to allow for a larger volumes in other dimensions of the experimental design, in particular, number of siRNAs tested and number of cells per siRNA. In other experiments, there may be analogous considerations that hinder tracking at the single-cell level, while still providing population-level time-course data.

In this study, we used a cell population-level dynamic model to represent the temporal evolution of dividing cells. By fitting cell counts in four transient cellular states, our model yielded parameters that quantify the dynamic effects of siRNA treatments on cell population levels. Model parameters allowed reliable estimation of the penetrance and time of four disruption events of the cell cycle: quiescence, mitosis arrest, polynucleation and cell death. We also derived the interphase and mitosis durations from penetrance parameters. We found 2190 siRNAs that resulted in quiescence, mitosis arrest, polynucleation or cell death at specific times, or increased interphase or mitosis duration. Comparison of the results with known cell-cycle and cell death regulators and systematic gene enrichment analysis indicate high sensitivity and accuracy of the method. The reported list is a useful resource, containing testable hypotheses about causal roles of genes in cell cycle regulation and cell death.

## Results and discussion

### Modelling cell population dynamics

**Figure 1:**
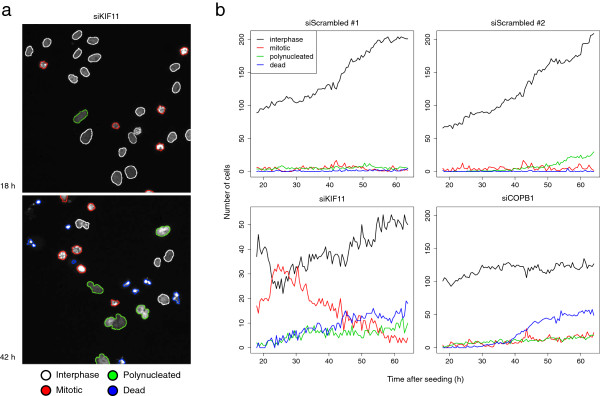
**Examples from the Mitocheck screen data.** **(a)** Images of HeLa cells imaged 18 h and 42 h after seeding on a control spot containing siKIF11, an siRNA targeting the kinesin gene KIF11. Interphase cells are outlined in white. Inhibition of KIF11 induced prometaphase arrest and led to an accumulation of mitotic cells (red). Of the arrested cells many subsequently became polynucleated (green) and eventually died (blue). **(b)** Time courses of cell counts in four spots. The negative control siRNA siScrambled led to normal cell growth in two replicated spots (top). siKIF11 led to an early accumulation of mitotic cells, while at later times many of the arrested cells went into cell death (bottom-left). siCOPB1, which targets an essential Golgi-binding protein, caused cell death, but no mitotic phenotypes (bottom-right). Y-axes scales were scaled to accommodate the different dynamic ranges of the data.

We considered the cell count data from the Mitocheck primary screen, consisting of 206,592 movies of siRNA spot experiments targeting 17,293 genes (Figure 1). Most of the genes (98.7%) were targeted by at least two independent siRNA sequences, each done in at least three spots. Four controls were repeatedly used on each slide: siScrambled, a non-targeting negative control; siKIF11, targeting the gene KIF11, which encodes a kinesin needed for centrosome segregation; siCOPB1, targeting an essential protein binding to the Golgi vesicle and siINCENP, targeting a centromere-associated protein coding gene required for proper chromosome segregation and cytokinesis. Each spot experiment yielded time courses of cell counts of 16 morphologically distinct transient nuclear morphologies, first acquired 18 h after cell seeding, and then measured every 30 min for 48 h. In total, more than 2 billion nuclear morphologies were measured and classified (Table 1).

**Figure 2:**
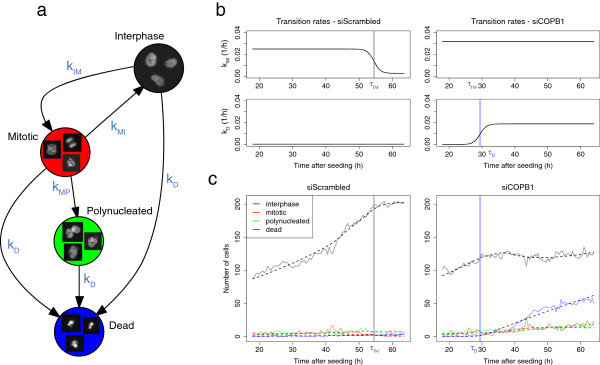
**A differential equation model to quantify temporal phenotypes.** **(a)** Cell populations were permitted to enter and leave states with four different transition rates: *k*_IM_, *k*_MI_, *k*_MP_ and *k*_D_. Representative images of cellular states are shown within the nodes. **(b)** Transition rates were allowed to vary over time, as modelled by parametric sigmoid functions. In the negative control spot (left), the interphase-to-mitosis transition rate *k*_IM_ inflected at *τ*_IM_ = 54.5 h, as the capacity of the spot to support a growing number of cells became limiting. The death transition rate *k*_D_ remained constant and null. In the siCOPB1 spot (right), the death transition rate *k*_D_ inflected at *τ*_D_ = 29.4 h, indicating the time of cell death. **(c)** Cell population time courses (light solid lines) and fitted data (dotted lines) of the spots described in panel b.

In order to quantify the phenotypic effect induced by siRNA treatments, we grouped the 16 nuclear morphologies into four cellular states recapitulating the cell cycle: interphase, mitotic, polynucleated and dead. We used an ordinary differential equation (ODE) model to characterise the dynamic transitions between the four populations (Figure 2a). We assumed that cells could enter and leave states with different, experiment-dependent transition rates. Among the twelve theoretically possible transitions between different states, we considered the six following ones: interphase cells may enter mitosis or die, mitotic cells may divide into twice as many interphase cells, become polynucleated or die, and polynucleated cells may die. We first considered a model with constant rates; however, we found that the data from many of the movies could not be fit satisfactorily. Therefore, we extended the model by allowing a simple time-dependence of the transition rates, motivated by the notion that the effect of an siRNA on a cell population occurs with a time delay after the transfection, reflecting differences in RNAi efficiency and protein life-time. Hence, to account both for experiment-dependent penetrance and delay of phenotypic effects, the transition rates were modelled with four parametric sigmoid functions (Figure 2b), each dependent on two parameters: a transition penetrance *α*_*x*_ and an inflection time point *τ*_*x*_. The same transition rate function *k*_D_(*t*) was used for all three transitions into cell death. The interphase-to-mitosis *k*_IM_(*t*) and mitosis-to-interphase *k*_MI_(*t*) transition rates were modelled with non-zero fixed intercepts, representing the basal rates in the untreated, proliferating populations. The model represents the temporal evolution of the four cell populations starting at cell seeding time, with an unknown initial number of cells *n*_0_. To account for normal cell contamination, resulting from untransfected cells moving into the spot region, we introduced an additional contamination parameter *μ* to represent the fraction of the cell subpopulation that follows a basal cell growth. Under this model, each spot experiment was described by 10 parameters: the initial number of cells *n*_0_ at seeding time, the contamination parameter *μ* and 8 transition parameters: penetrance *α*_*x*_ and inflection time *τ*_*x*_ each for *k*_D_(*t*),*k*_IM_(*t*),*k*_MI_(*t*) and *k*_MP_(*t*).

For each spot experiment, parameters were robustly estimated by fitting the cell count time course to the model by penalised least squares (Figure 2c). The mean relative error (MRE), i. e., the average of absolute differences between the fitted and the measured cell counts relative to the maximum number of cells, measured the accuracy of the fit in one spot. 95% of the spot experiments had an MRE lower than 3.2%, demonstrating the overall high goodness of fit of the model. Spot experiments with high MRE, indicative of lack of model fit, were discarded from the analysis. We visually inspected a random selection of about 10 of these movies and associated time courses, and in all cases, technical artefacts such as loss-of-focus, spotting issues or well contamination were identified as source of the misfit.

### Analysis of siRNAs disrupting the cell cycle

In normal exponential growth, cells are transitioning from interphase to mitosis and back to interphase at constant rates. We focused on four types of disruptions of the basal cell cycle shown in Figure 2: quiescence, when cells stop dividing, mitotic arrest, when cells stop going back in interphase, polynucleation, when cells start becoming polynucleated and cell death, when cells start to die. Each of these events was associated with a corresponding transition penetrance and inflection time.

**Figure 3:**
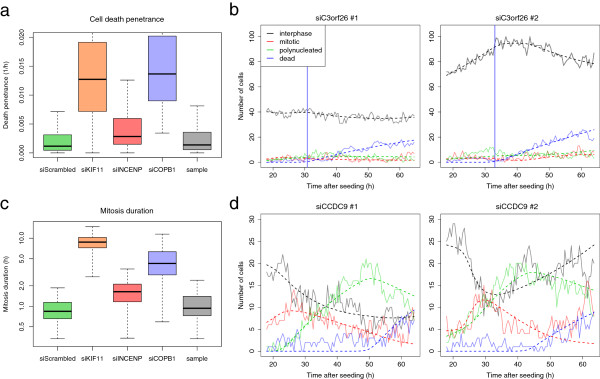
**Model fits provided meaningful biological parameters that helped annotate gene function.** **(a)** Box plots of cell death penetrance *α*_D_ estimated in control and sample wells. Lethal phenotypes induced by siKIF11 and siCOPB1 had significantly higher cell death penetrance than the negative siScrambled control spots. **(b)** Cell count time courses of two spots containing an siRNA (MCO_0026491) targeting the not well-characterised gene C3orf26. Induction of cell death started at *τ*_D_ = 30.8 h and 33.8 h. **(c)** Box plot of mitosis duration estimated in control and sample wells. Prometaphase arrest caused by siKIF11 led to a significantly higher mitosis duration than cells in negative control spots. **(d)** Cell count time courses of two spots containing an siRNA (MCO_0020444) targeting the poorly characterised gene CCDC9. The overall high proportion of mitotic cells, compared to the negative controls illustrated in Figure 1b, is indicative of a longer mitosis duration, estimated at 5.7 h.

Transition penetrances proved to be reliable indicators of disruptions of the cell cycle. As an example, in cell death, cells growing in the control spots siCOPB1 had a significantly higher mean cell death penetrance (1.63 × 10^-2^ h^-1^) than cells seeded in the negative control spots (0.29 × 10^-2^ h^-1^, Wilcoxon rank sum test, P < 10^-15^) (Figure 3a). This is in agreement with the essential role of COPB1 in binding Golgi vesicles. Similarly, cells subject to siKIF11 had a significant higher mean mitotic arrest penetrance than negative control spots (5.58 × 10^-1^ versus 0.27 × 10^-1^ h^-1^, P < 10^-15^) and a high mean cell death penetrance (1.40 × 10^-2^ h^-1^), consistent with cell death that follows prometaphase arrest induced by the treatment. Based on these observations, we defined thresholds on each of the four transition penetrances to detect the siRNAs that disrupt the cell cycle (see Methods).

Transition inflection points quantified the times of disruption of the cell cycle. For each siRNA, we summarised the four times obtained from the replicate spots by average and standard deviation. We identified genes with reproducible cell cycle disruption times by requiring standard deviation of less than 4 h and average of less than 50 h after seeding time; the latter criterion was motivated by the generally lower confidence of the inflection time estimates at later times. Using these criteria, we found 168 siRNAs leading to quiescence at reproducible times, 289 inducing mitotic arrest, 390 leading to polynucleation and 171 causing cell death Additional file 1: Table S1).

The targets of the siRNAs inducing cell death included the protein units of the Golgi vesicular coat COPA and COPB2, several known apoptosis regulators such as TP53AIP1 and the RAS family members RAB25 and RAN. Interestingly, three siRNAs targeting COPA and COPB2 induced cell death at similar time points (34.4 h, 35.3 h and 37.5 h), together with siCOPB1 (31.8 h). The similarity of these timings is consistent with the fact that the proteins are part of the same protein complex. On the contrary, siRNAs directed at the RNA helicase DDX39A induced an early cell death at 22.8 h, which could reflect a different cell death mechanism from the one caused by COPA and COPB2 inhibition. We also identified several siRNAs inducing cell death and targeting uncharacterised genes such as C3orf26 (Figure 3b), C3orf52 or C16orf90. However, due to the existence of off-target effects in RNA interference, functional rescue of the phenotypes and secondary functional assays would be needed to confirm the essential role of these genes.

We found 289 siRNAs inducing mitotic arrest at reproducible times, including the cyclin-dependent kinase inhibitor CDKN2A at 26.1 h, the cell cycle progression control protein CDC40 at 36.2 h or NEK2, a kinase involved in the control of centrosome separation and bipolar spindle formation, at 48.2 h. Due to the coupled nature of mitotic arrest and cell death that may follow, we analysed the 36 siRNAs that induced these two phenotypes at reproducible times in Additional file 2: Figure S1. As expected, Pearson correlation between time of mitotic arrest and time of cell death was 0.80, confirming the relationship between the phenotypes.

### Analysis of siRNAs increasing mitosis and interphase duration

Average residence time in a cellular state can be derived from transition penetrances using dimensional arguments, as described in the Methods section. In particular, we were able to estimate mitosis duration (Figure 3c) and interphase duration from the model parameters. Cells growing in negative control spots had a median mitosis duration parameter of 51 min, in agreement with live imaging studies in HeLa cells [11]. In contrast, for cells treated with siKIF11 the value for this parameter was strongly elevated to 8.8 h (Wilcoxon rank sum test, P < 10^-15^), consistent with the essential role of KIF11 in progression to metaphase. Similarly, for cells treated with siINCENP the mitosis duration parameter was 1.6 h (P < 10^-15^), reflecting the need of INCENP for proper chromosome segregation.

We summarised the mitosis duration parameter for each siRNA by computing the geometric mean of the values from the replicate spots. The geometric mean was chosen over the arithmetic mean to reduce the influence of outliers from highly variable large mitosis duration estimates. We ruled that siRNA mitosis duration could not be reliably estimated when the geometric standard deviation, i. e. the exponentiated value of the standard deviation of the log transformed values, of the replicate spots was higher than 2 h. We found 1251 siRNAs, targeting 1190 unique genes, that increased mitosis duration to more than 2 h, two times the basal mitosis duration (Additional file 1: Table S1). Gene ontology (GO) enrichment analysis of the target genes showed significant enrichment of mitotic cell cycle regulation processes (Table 2). Many known genes involved in mitosis progression were found, including the mitogen-activated protein kinases MAP2K4 and MAP3K2, two subunits of the anaphase promoting complex ANAPC1 and ANAPC4, the M-phase phosphoprotein MPHOSPH6 and the cell cycle regulating kinases NEK2, NEK9 and NEK10 [12]. Many siRNAs targeting protein-coding genes with unknown functions were found, including C12orf5, C3orf32 and CCDC9 (Figure 3d). As an example, targeting the coiled-coil domain containing gene CCDC9 caused cells to undergo mitosis in about 5.7 h. This result suggests that CCDC9 may be required for mitotic progression, and it will be interesting to further investigate such candidates in validation experiments.

**Table 2:** Gene ontology terms enriched in the 1251 siRNAs increasing mitosis duration

	**Term**	**p.value**	**Odds.ratio**	**Annotated**	**Hit**	**Description**
1	GO:0051437	8.7 × 10^-12^	7.43	70	24	Positive regulation of ubiquitin-protein ligase activity involved in mitotic cell
						cycle
2	GO:0032270	1.6 × 10^-7^	2.36	391	55	Positive regulation of cellular protein metabolic process
3	GO:0072413	2.3 × 10^-7^	5.60	60	17	Signal transduction involved in mitotic cell cycle checkpoint
4	GO:0000216	7.9 × 10^-7^	4.72	72	18	M/G1 transition of mitotic cell cycle
5	GO:0031570	9.1 × 10^-7^	3.59	119	24	DNA integrity checkpoint
6	GO:0008380	2.2 × 10^-6^	2.41	291	42	RNA splicing
7	GO:0007093	3.8 × 10^-6^	3.36	120	23	Mitotic cell cycle checkpoint
8	GO:0071843	1.1 × 10^-5^	2.59	201	31	Cellular component biogenesis at cellular level
9	GO:0000075	2.8 × 10^-5^	2.50	207	31	Cell cycle checkpoint

Shown is a selection of GO terms that were significantly over-represented (Fisher’s exact test) with a p-value lower than 10^-4^. Most of the terms are related to mitosis and cell cycle regulation.

Similar to mitosis duration, we found 288 siRNAs that increased interphase duration to more than 40 h, with a geometric standard deviation lower than 4 h (Additional file 1: Table S1). GO enrichment analysis of the 286 unique targets revealed a significant enrichment of genes coding for proteins involved in metabolic processes of amines, carboxylic acids and alcohols (Fisher’s exact test, P < 5 × 10^-4^). Perturbations of the metabolism of fast-growing cells are a plausible reason for decelerated cell growth and hence for an increase of interphase duration.

### Clustering phenotypes

The fitted transition parameters quantified the phenotypic effect of a treatment on a cell population in a multivariate manner. The parameters were designed to not depend on the initial number of cells at seeding time or on contamination by untransfected cells moving into the spot region. Moreover, the penetrance parameters were time independent and unaffected by possible delays that could have occurred during slide preparation. As a result, most of the variability due to cell seeding, siRNA spotting or delays in plating should have small influence on the parameter estimates. Therefore, our model can be seen as a efficient method to estimate the phenotypic effect of a treatment on a cell population, separating the biological signal from the technical variability coming from the assay.

**Figure 4:**
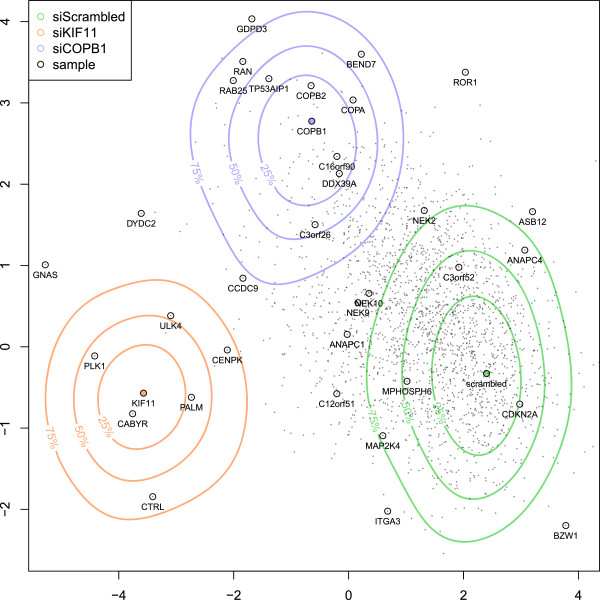
**Phenotypic map of the Mitocheck screen.** Phenotypic profiles of all siRNA treatments were derived from fitted parameters and projected into a two dimensional space using linear discriminant analysis between the control spots siScrambled, siCOPB1 and siKIF11. Color contour lines denote the quantiles of control experiments that fall into the corresponding phenotypic region. The green phenotypic region, centered on the non-targeting negative control siScrambled, is representative of a normal growth phenotype. The orange phenotypic region, centered on siKIF11, is representative of prometaphase arrest followed by cell death, while the blue region, centered on siCOPB1, is representative of cell death without mitotic arrest. Shown are the 2190 siRNAs that induced a disruption of the cell cycle, or increased the duration of the interphase or mitosis phases. A selection of siRNA gene targets are highlighted on the map.

To generate a phenotypic profile for each siRNA, we used the inflection time parameters and the logarithm-transformed penetrance parameters and summarized measurements from multiple spots per siRNA by the median. Phenotypic profiles were projected in two dimensions using linear discriminant analysis between the siScrambled, siCOPB1 and siKIF11 control spots (Figure 4). The projection recapitulated many of the previous findings: the vesicular coat protein-coding genes COPA, COPB1 and COPB2 clustered in the same region, characterised by cell death. The kinase genes NEK9 and NEK10 also clustered together, characterised by a complex phenotype dominated by mitosis defect, polynucleation and cell death. C3orf26 fell into a phenotypic region dominated by cell death, while CCDC9 was located between siCOPB1 and siKIF11, consistent with their phenotypes observed in Figure 3. Similar to the approach used in [6], genes with similar phenotypic profiles are frequently functionally related, and further studies can be performed to annotate the function of uncharacterised genes.

## Conclusions

Time-lapse data can provide more information than endpoint assays. For instance, the endpoint *cell death* can be reached by different avenues, and intermediate phenotypes, such as mitotic arrest, that precede the eventual outcome provide important information on mechanistic or causal specifics of the final outcome. We have presented a population-based modelling approach to quantify dynamic phenotypes from time-lapse cell imaging assays. The temporal information helps to localise the timing of events such as cell death, mitotic arrest or quiescence, and to estimate the duration of processes such as mitosis.

Our approach models the temporal evolution of the population size of cellular states by a system of ODEs. This choice was motivated by the fact that sufficiently accurate tracking information on individual cells was not available for these data. It is possible to interpret the ODE model as an approximation of the time evolution of the mean cell numbers (expectation values) of an underlying stochastic Markov process in the discrete space of cell state frequencies, from which it emerges by Ω-expansion of the master equation [13]. For the population sizes and transition types and rates of interest here, the approximation holds well, and effects of the discrete or stochastic nature of such a process on the evolution of the means [14] is expected to be negligible compared to the experimental variability of the data. However, if tracking information had been available, then using it might have given more direct results, e. g., on residence time distributions of the cells in the different states. Due to the presence of cell death and cell division, tracking needs to be integrated with the model fitting of a suitably defined stochastic process. An example of such an approach was presented in the CellCognition methodology [10].

We used a 10-parameter ODE model with 4 cellular states and 4 independent transition rates. We selected this model based on the following criteria: complexity of the model, goodness-of-fit, parameter identifiability and biological significance of the parameters. We were able to fit our model on the vast majority of spot experiments, demonstrating its overall high goodness-of-fit, despite the broad variety of dynamic phenotypes of the Mitocheck assay, the overall low cell counts, the cell misclassification noise and the presence of untransfected cells. At the same time, we were able to reliably estimate the 10 model parameters with satisfactory precision, as is indicated by the reproducibility between the control spots, shown in the clear separation of control phenotypes in Figure 4. As part of the model development, we tested simpler and more complex models. The models with fewer parameters, however, failed to model the complex phenotypes of some of our positive controls, such as siKIF11 (data not shown). Parameter identifiability was a problem in more complex models, e. g., when allowing three separate cell death transition rates, or two different polynucleated states. In these models, some parameters could not be reliably estimated due to low cell counts and cell misclassification noise, and they were often shrunk to zero due to the penalized estimation procedure. Our model was primarily designed to quantify the phenotypes of a large-scale imaging assay with relatively low temporal resolution and showing a broad variety of dynamic phenotypes. Depending on the biological question, more targeted models could be envisioned to focus on certain dynamic aspects, such as introducing different modes of cell death or using a finer model of the mitosis phase.

We applied our model to the Mitocheck assay and derived six new phenotypic descriptors not considered in the original analysis: time of quiescence, time of mitotic arrest, time of polynucleation and time of cell death, mitosis duration and interphase duration. We established a list of 2190 siRNAs where these phenotypes could be reliably estimated. This list can be seen as a resource to build new hypotheses on the associations between genes and biological processes. However, due to the possibility of off-target effects of siRNA perturbations, unavoidable experimental variability and the use of a cell line with a heavily rearranged genome, for general validity these results must be confirmed by independent assays, for instance, rescue experiments in another cell line [8].

## Methods

### The Mitocheck time-lapse imaging screen data

We used the Mitocheck primary screen data available from http://www.mitocheck.org, an online database containing the siRNA sequences, movies and cell count times courses. The screen consisted of 206592 time-lapse spot experiments, including 164875 sample spots, using 51766 different siRNA constructs and targeting 17293 genes (based on a mapping to the Ensembl version 27 annotation of the human genome). Spot experiments were organised in slides, each containing 7 to 8 negative and 8 to 11 positive control spots. A cervix carcinoma cell line (HeLa) that stably expresses GFP-tagged H2B histone was used for fluorescence microscopy. Cells were filmed starting 18 h after seeding for a duration of 48 h, with an imaging rate of one per 30 min. After acquisition, cell nuclei were segmented, quantified and classified into one of 16 morphological classes by a fully automated algorithm [8]. We grouped the 16 nuclear morphologies into 4 cellular states representative of the cell cycle: “interphase”, “mitosis”, “polynucleated” and “cell death” (Table 1).

### Ordinary differential equation model

We considered four cellular states: interphase (*I*), mitotic (*M*), polynucleated (*P*) and dead (*D*). We modelled the number of cells *n*_*p*_(*t*) of state *p*, at time *t* with the system  of differential equations, depicted in Figure 2a:

$$\begin{array}{lll} \;{\dot{n}}_{\text{I}}(t) & =-\left(k_{\text{IM}}(t)+k_{\text{D}}(t)\right)n_{\text{I}}(t)+2k_{\text{MI}}(t)n_{\text{M}}(t)\; & \;\\ \;{\dot{n}}_{\text{M}}(t) & =k_{\text{IM}}(t)n_{\text{I}}(t)\; & \;\\ & \;-\left(k_{\text{MI}}(t)+k_{\text{D}}(t)+k_{\text{MP}}(t)\right)n_{\text{M}}(t)\; & \;\\ \;{\dot{n}}_{\text{P}}(t) & =k_{\text{MP}}(t)n_{\text{M}}(t)-k_{\text{D}}(t)n_{\text{P}}(t)\; & \;\\ \;{\dot{n}}_{\text{D}}(t) & =k_{\text{D}}(t)n_{\text{I}}(t)+k_{\text{D}}(t)n_{\text{M}}(t)+k_{\text{D}}(t)n_{\text{P}}(t),\; & \; \end{array}$$

with:

$$\begin{array}{lll} \;k_{\text{IM}}(t) & =\alpha_{\text{IM}}^{0}-\alpha_{\text{IM}}/\left(1+\exp(\tau_{\text{IM}}-t)\right)\; & \;\\ \;k_{\text{MI}}(t) & =\alpha_{\text{MI}}^{0}-\alpha_{\text{MI}}/\left(1+\exp(\tau_{\text{MI}}-t)\right)\; & \;\\ \;k_{\text{MP}}(t) & =\alpha_{\text{MP}}/\left(1+\exp(\tau_{\text{MP}}-t)\right)\; & \;\\ \;k_{\text{D}}(t) & =\alpha_{\text{D}}/\left(1+\exp(\tau_{\text{D}}-t)\right)\; & \; \end{array}$$

where ${\dot{n}}_{p}(t)$ is the time derivative of *n*_*p*_(*t*), with the initial conditions *n*_I_(0) = (1-*ω*_0_)*n*_0_, *n*_M_(0) = *ω*_0_*n*_0_, *n*_P_(0) = *n*_D_(0) = 0, and *n*_0_ is the number of cells at seeding time *t* = 0. In agreement with observations of untreated cells, the mitotic index at seeding time was set to *ω*_0_ = 0.05, the basal interphase-to-mitosis penetrance to $\alpha_{\text{IM}}^{0}=0.025$ h^-1^ and the basal mitosis-to-interphase penetrance $\alpha_{\text{MI}}^{0}=0.57$ h^-1^. To account for contamination of spots by normal cells, due to untransfected cells moving into the spot region, we assumed that cell counts were a mixture of two independent, growing cell populations: a treated population, modelled by  with parameters {(1-*μ*)*n*_0_, *α*_IM_, *α*_MI_, *α*_MP_, *α*_D_, *τ*_IM_, *τ*_MI_, *τ*_MP_, *τ*_D_} and an untreated population, modelled by  with parameters {*μn*_0_, 0, 0, 0, 0, 0, 0, 0, 0}. In total, 10 parameters were required to model a cell count time course: the initial cell number at seeding time *n*_0_, the normal contamination fraction parameter *μ* and 8 transition parameters {*α*_IM_, *α*_MI_, *α*_MP_, *α*_D_, *τ*_IM_, *τ*_MI_, *τ*_MP_, *τ*_D_}.

### Estimation of model parameters

Model parameters *θ* = {*n*_0_, *μ*, *α*_IM_, *α*_MI_, *α*_MP_, *α*_D_, *τ*_IM_, *τ*_MI_, *τ*_MP_, *τ*_D_} were estimated by penalised least squares regression, minimising the cost function:

(1) $$J(\theta )=\frac{1}{\#T}\underset{t\in T}{\sum }\underset{p\in P}{\sum }{\left(y_{p}(t)-n_{p}(t,\theta )\right)}^{2}+\lambda \underset{k\in K}{\sum }\alpha_{k}^{2},$$

where  is the set of observed time points, *y*_*p*_(*t*) the observed number of cells of state *p* at time *t*, *λ* a constant to weigh the penalty term and the set of penalised parameters $K=\{\mu ,\;\alpha_{\text{IM}},\;\alpha_{\text{MI}},\;\alpha_{\text{MP}},\;\alpha_{\text{D}}\}$. Given the parameters, the ODE system was integrated using the Runge-Kutta fourth-order method. Minimisation of the penalised criterion *J* was achieved with the Levenberg-Marquardt algorithm [15], applying a positivity constraint to the components of *θ*. To decrease the risk of finding local minima, each numeric minimisation was run 64 times with different initial parameters that were randomly sampled from previous fitted parameters with Gaussian noise added. Using 64 initial conditions greatly decreased the variance of the estimated parameters, as shown in the Additional file 3: Figure S2. The regularisation parameter *λ* was selected to maximise the classification performance of the model parameters on the data subset of control siRNAs, computed by 5-fold cross-validation and linear discriminant analysis. We chose the value *λ* = 4, leading to a classification performance of 94.2%. The regularisation did not substantially alter the overall goodness-of-fit in terms of residual sum of squares. The penalty term accounted, on average over all spots, for 6.3% of the cost function (1).

The associated software, which is designed to also be applicable to analogous time-lapse cell count data, and the code to reproduce the results shown in this paper, are available in the Bioconductor/R package *mitoODE*.

### Data transformation and quality control

For the estimation of the time of disruption of the cell cycle, we selected those spot experiments that had sufficiently high transition penetrance parameters {*α*_IM_, *α*_MI_, *α*_MP_, *α*_D_}. That is, we required these parameters to be higher than 0.025, 0.5, 0.25 or 0.005, respectively. These values corresponded approximately to the 90% quantile of the corresponding values in the negative control (siScrambled) spots. An example of the distribution of the cell death penetrance is shown in Figure 3a.

Dimensional arguments show that the mean residence time in a cellular state is proportional to the reciprocal of the output transition rate. Therefore, we defined the mean mitotic duration as *γ*_*M*_/(*α*_MP_ + *α*_D_-*α*_MI_) and the mean interphase duration as *γ*_*I*_/(*α*_D_-*α*_IM_), where *γ*_*M*_ and *γ*_*I*_ are experiment-specific parameters. Average mitosis duration of 50 min and interphase duration of 23 h are observed in untreated HeLa cells in higher-resolution live cell imaging experiments, and based on these values we calibrated *γ*_*M*_ = 0.6 and *γ*_*I*_ = 1.

To account for experimental artefacts such as accumulation of cell clusters, transient loss-of-focus, spot contamination and seeding issues, we implemented a quality control (QC) filter. We disregarded all spots that did not pass the the original Mitocheck spot QC filter [8], had a cost value of *J* (Equation (1)) higher than the 95% quantile of all *J*, had a normal contamination fraction *μ* higher than 0.5, or had an estimated mitosis duration of less than 20 min, which is biologically implausible. In total, 83.4% of the 164875 sample spot experiments passed the quality filter. Visual inspection confirmed that discarded spots were mostly experimental artefacts.

## Competing interests

The authors declare that there are no competing interests.

## Authors’ contributions

GP and WH designed the method and wrote the manuscript. GP, TW and JKH analysed the results. GP, TW, BN, JKH and JE contributed to the biological interpretation of the results. All authors read and approved the final manuscript.

## Supplementary Material

Additional file 1 **Table S1.** List of 2190 siRNAs inducing a disruption of the cell cycle. The “Mitocheck.sirnaID” column indicates the siRNA IDs, as referenced in the http://www.mitocheck.org database. The “target.hgnc“ column indicates the HUGO gene symbol targeted by the siRNAs. The four next columns are the different times of disruption of the cell cycle induced by the siRNAs, in hours. The two next columns are the measured durations of the interphase and mitosis phases induced by the siRNAs, in hours. Click here for file

Additional file 2 **Figure S1.** Correlation between time of mitotic arrest and time of cell death. Pearson’s correlation between the time of mitotic arrest and the time of cell death measured in 36 siRNAs inducing both phenotypes was 0.80, confirming the relationship between the phenotypes. Click here for file

Additional file 3 **Figure S2.** Effect of multiple initial conditions in parameter estimate variance. The variance of the estimate of the mitosis-to-interphase *α*_*MI*_ penetrance rate in the siKIF11 spot shown in Figure 1b (bottom-left) depends of the number of initial conditions used in the fitting procedure. For each of the boxes shown, we estimated 32 times the penetrance rate *α*_*MI*_ of the spot, keeping the lowest cost estimate from different numbers of initial conditions (1, 4, 16 and 64), randomly sampled from previously fitted spots. As expected, the variance of the estimates decreased greatly with increasing number of initial conditions. The horizontal red line shows the final estimate used in the analysis. Click here for file
