# Supplementary figures and images for: Dynamical modelling of phenotypes in a genome-wide RNAi live-cell imaging assay
Source: BMC Bioinformatics. 2013 Oct 16;14:308. doi: 10.1186/1471-2105-14-308 (PMC3827932; doi:10.1186/1471-2105-14-308)

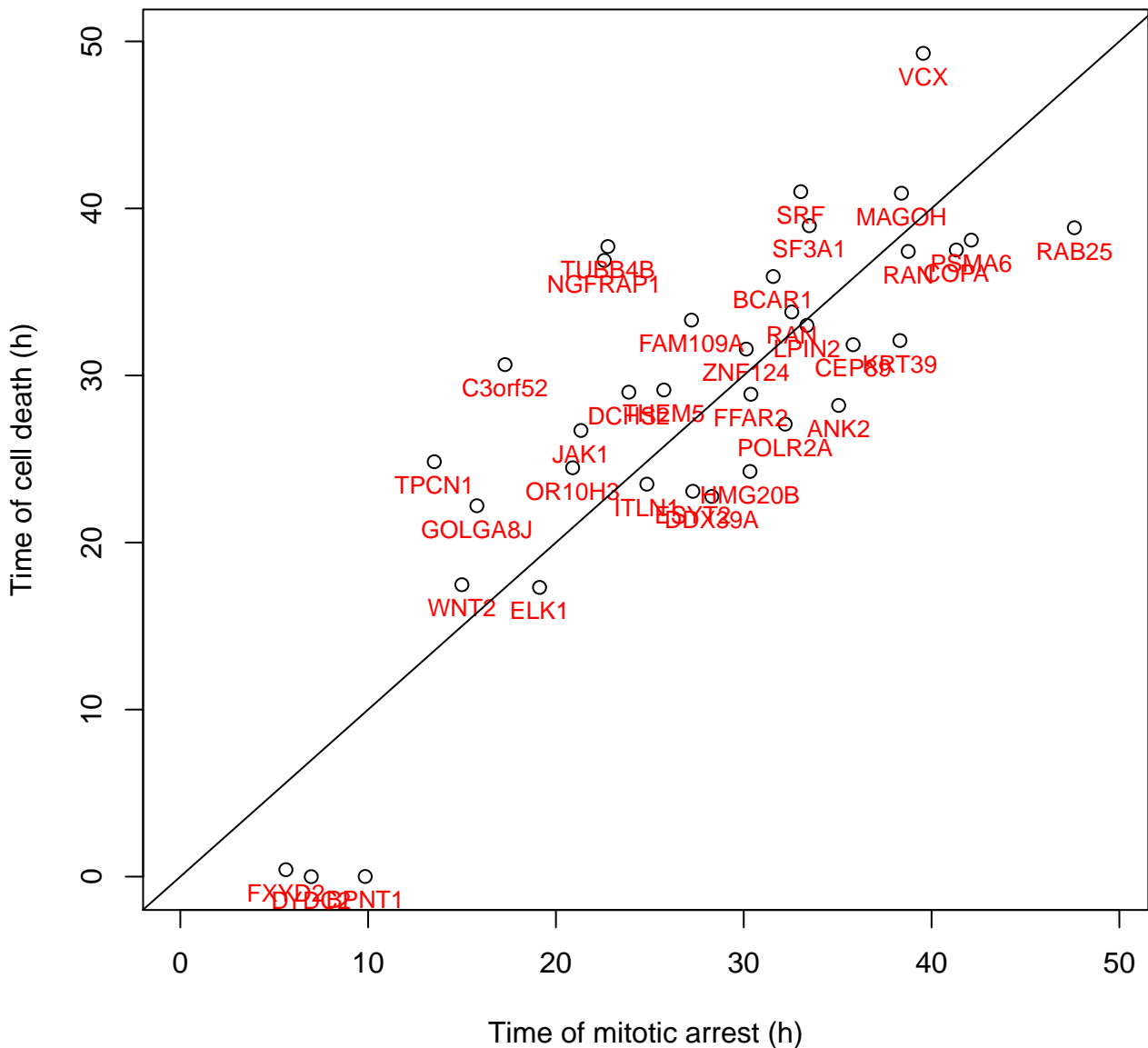

Supplement: Additional file 2 — Figure S1. Correlation between time of mitotic arrest and time of cell death. Pearson’s correlation between the time of mitotic arrest and the time of cell death measured in 36 siRNAs inducing both phenotypes was 0.80, confirming the relationship between the phenotypes. [file 1471-2105-14-308-S2.pdf]

# Model fitting of siKIF11

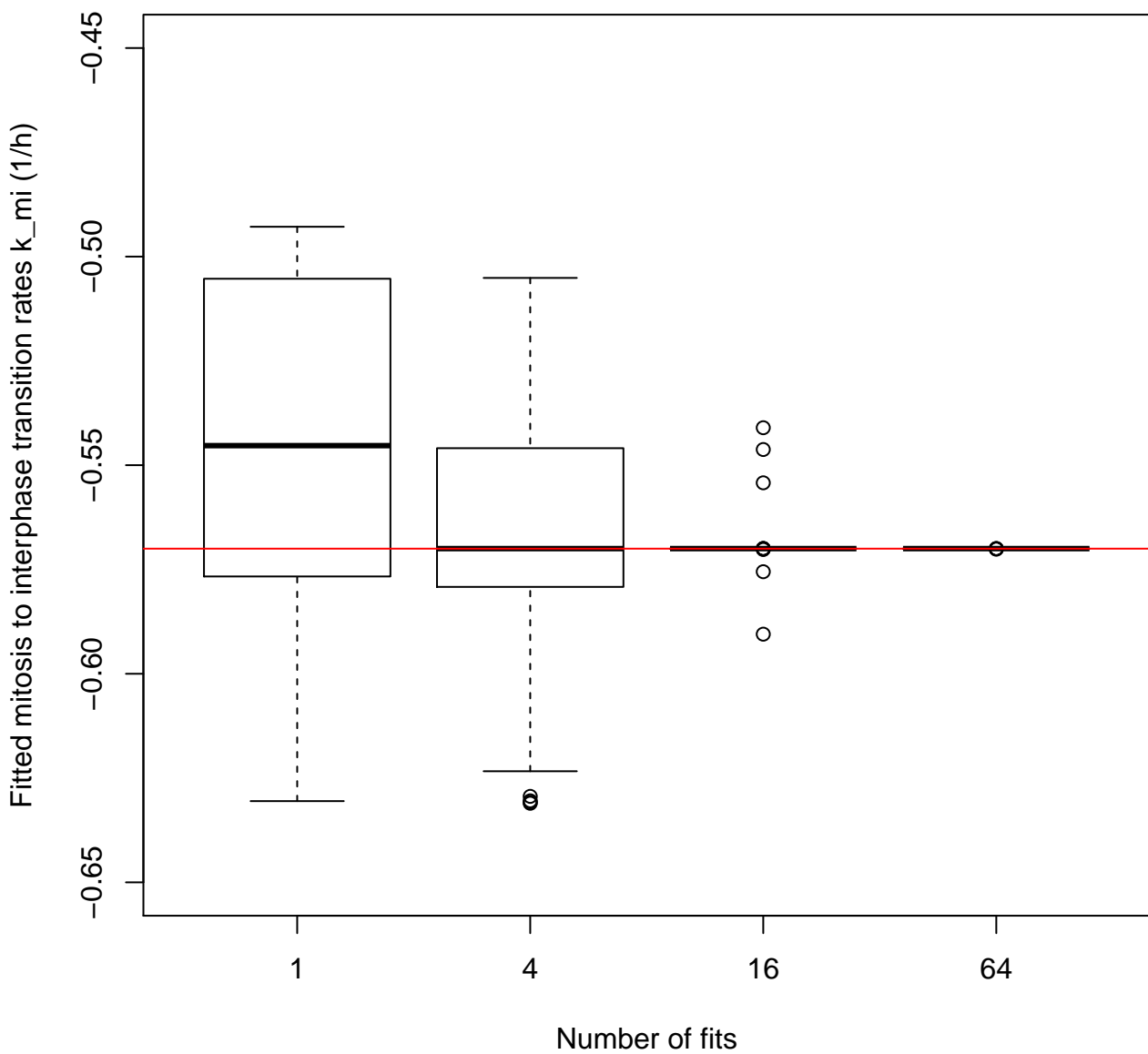

Supplement: Additional file 3 — Figure S2. Effect of multiple initial conditions in parameter estimate variance. The variance of the estimate of the mitosis-to-interphase αMI penetrance rate in the siKIF11 spot shown in Figure 1b (bottom-left) depends of the number of initial conditions used in the fitting procedure. For each of the boxes shown, we estimated 32 times the penetrance rate αMI of the spot, keeping the lowest cost estimate from different numbers of initial conditions (1, 4, 16 and 64), randomly sampled from previously fitted spots. As expected, the variance of the estimates decreased greatly with increasing number of initial conditions. The horizontal red line shows the final estimate used in the analysis. [file 1471-2105-14-308-S3.pdf]
